# Supplementary material for: Comparative analysis of diagnostic methods for the detection of Cryptococcus neoformans meningitis
Source: PLoS Negl Trop Dis. 2023 Mar 6;17(3):e0011140. doi: 10.1371/journal.pntd.0011140 (PMC10019727; doi:10.1371/journal.pntd.0011140)
Supplement: Attachment — Submitted filename: PLOS NTD article Reading Report.docx [file pntd.0011140.s001.docx]

**Article Reading Report named**:

« **Comparative analysis of diagnostic methods to detection *Cryptococcus neoformans* meningitis** »

Very informative article on the comparative analysis of diagnostic methods to detection of *Cryptococcus neoformans* meningitis, mainly in people living with HIV.

In the following lines, I pass on observations that I believe will help improve the quality of this article.

I. **Major comments**

- The superiority of molecular techniques over conventional techniques is already established in the literature. However, the proposal of early diagnostic tests based on the analysis of the *Cryptococcus* genetic material seems to me more original and relevant. It is therefore preferable to put this aspect first. This will involve adaptations of some parts of the manuscript.

II. **Minor comments**

- There are no line numbers in the manuscript. This makes it difficult to track observations.
- In the context of identification/characterization, it is better to refer to *Cryptococcus* rather than cryptococcosis (throughout all the text). Although it is also usable.

Abstract

- In the introduction, it seems to me that a sentence is missing explaining the gap that the study intends to fill.
- Methodology >>> Methods
- The rapid India ink >>> The direct India ink staining (methods)
- Please provide comparative values for sensitivity and specificity of tests (results).
- The comparative aspect of the tests proposed as an objective of the manuscript is not clearly expressed in the conclusion.

Here a proposal: “Molecular techniques, in particular nested PCR specific to the 5.8Sr DNA sequence, show a better detection of *Cryptococcus* infection than conventional detection methods and 18S PCR, both in serum and CSF. They also allow a better early identification in case of negative serology and mycology, potentially allowing an early diagnosis”.

Introduction

- New data on the global burden of cryptococcosis in HIV patients were recently published in 2022 by Radha Rajasingham *et al*. https://doi.org/10.1016/ S1473-3099(22)00516-3. Please update
- Studies on systemic mycosis have shown that cryptococcosis is the second leading cause of AIDS-associated death from systemic mycosis in Brazil **>>>** after which opportunistic infection?
- The diagnosis of cryptococcosis is complex and **~~is~~** usually based on a combination of clinical and radiological suspicion followed by confirmation by laboratory diagnosis
- In patients with HIV-associated infections, culture of cerebrospinal fluid (CSF) is the gold standard for diagnosis of cryptococcal meningitis in **viable sample…**

Methods

- Although it is of little importance, I would not begin this section with ethical considerations.
- Serum and CSF from the same patient? Two types of samples per patient? Please specify –
- The study was approved by the institutional ethics committees of **the** Hospital das Clinicas da Faculdade de Medicina da Universidade de Sao Paulo - HCFMUSP (No. 0635/2007) and **the** Instituto de Infectologia Emílio Ribas - IIER (No. 63/2007).
- Patients >>> patients and samples
- After initial isolation in culture subsequent identification was by physiological and biochemical tests as described below: is it possible to quote at least the tests used?
- and AIDS/HIV-positive and positive for cryptococcosis (group IV; n=18) >>> and **~~AIDS/~~**HIV-positive and positive for cryptococcosis (group IV; n=18)
- (600-base pair [bp] product) and 18S 9429-bp product) >>> [600-base pair (bp) product] and 18S (9429-bp product).
- Multiplex PCR: *Cryptococcus spp* >>> *Cryptococcus* spp
- On the basis of the drawbacks of conventional routine techniques as notified in the introductory part, would you have the possibility to integrate the detection of inter-ribosomal DNA in the daily practice?
- The significance level was set at p ≤ 0.05: please make the font uniform.

Results

- How were non-cryptococcal agents identified in the samples? other fungi, bacteria and viruses?
- Table 2: Order of presentation of parameters = underlying diseases first, then HIV status.
- Prior to the *Cryptococcus* spp. assay, patient samples were subjected to nested PCR against the housekeeping gene GADPH, confirming in all samples the presence of human DNA and the absence of PCR inhibitors: This seems redundant to me because it has already been mentioned in the method section (page 10 - 11).

Also, the interpretive explanations of the Kappa test should be in the methods, they are not results.

- All assays were performed in triplicate and showed 100% reproducibility. **I**n addition, …
- It is essential to use the new nomenclature of *Cryptococcus* species: *Cryptococcus var grubii* >>> *C. neoformans*. This is for the whole manuscript
- The 18S PCR assay results in HIV-positive patients were similar for both sera and CSF~~,~~ (Kappa index of 0.566 and 0552, respectively),
- However, when testing CSF, the 18S PCR exhibited a greater accuracy than **~~did~~** the latex agglutination test
- A comparative analysis of the 18SDNA primers with the latex agglutination assay for sera yielded a similar specificity and sensitivity, suggesting that the latex agglutination assay can be used as a ~~complementary~~ alternative test to this PCR test
- The comparative analysis between conventional (~~mycological~~ India ink staining and serological)
- Overall, I note that the proportions compared by test are not presented. This could improve the understanding of some paragraphs.

Discussion

- The increased availability of anti-retroviral treatment (ART) plus aging of the HIV population may have contributed to changes in the spectrum of disease manifestation, including complications that may be attributable to the neurotoxic consequences of ART >>> I'm having trouble finding the connection of this sentence to the previous sentences.
- In addition, false-positive results in sera and CSF from HIV-positive and ~~HIV~~- negative patients can occur under various conditions, such as in the presence of rheumatoid factors or infections with *Trichosporon beigelii*, *Klebsiella pneumonia*, *Capnocytophaga canimorsus* or *Stomatococcus mucilaginosus*~~.~~ [20, 59].
- A comparative analysis of the 18SDNA primers with the latex agglutination assay for sera yielded a similar specificity and sensitivity, suggesting that the latex agglutination assay can be used as a ~~complementary~~ alternative test to this PCR test
- The lower sensitivity and specificity with the 18S primers in the CSF samples may be due to interference in gene amplification by components of the Cryptococcus spp. cell wall or the antiretroviral treatment >>> How do you explain the particularity of these two parameters at the level of 18S primers
- Multiplex PCR and sequencing data have confirmed that 95-100% of C. neoformans biological samples from AIDS patients worldwide, including in Brazil, are from the *grubii* strain (serotype A) >>> please consider the new nomenclature
- The present study has limitations that must be acknowledged. The patients’ medical records did not contain all relevant information, such as occupational risk factors. In addition, the analysis of patients’ follow-up was limited to their hospitalization and so the risk of recurrence or other complications after discharge could not be assessed and the small??? >>> truncated sentence?
- Although further studies are needed, our results indicate that the combination of nested 5.8S PCR testing ~~in association~~ with the latex agglutination test may increase the ability to diagnose and monitor cryptococcosis in patients.
