# Supplementary material for: Comparative analysis of diagnostic methods for the detection of Cryptococcus neoformans meningitis
Source: PLoS Negl Trop Dis. 2023 Mar 6;17(3):e0011140. doi: 10.1371/journal.pntd.0011140 (PMC10019727; doi:10.1371/journal.pntd.0011140)
Supplement: Attachment — Submitted filename: Cover letter with Point-by-point response to reviewers.docx [file pntd.0011140.s002.docx]

São Paulo, January 12^th^, 2023

Dear Marcio L. Rodrigues,

Section Editor

PLOS Neglected Tropical Diseases

Ref. submission PNTD-D-22-01273 – [EMID:bcd15d4fa66f0da4]

# "Comparative analysis of diagnostic methods for the detection of *Cryptococcus neoformans* meningitis”

Thank you for giving us the opportunity to revise our manuscript. Please find enclosed the answers to the comments made by the reviewers. For the sake of clarity, we have submitted a point-by-point reply to the reviewers, and all modifications made in the revised text are highlighted in red. We hope that we have clearly addressed all of the questions raised by the reviewers and that our work, in its present form, has reached the high standards for publication in PLOS Neglected Tropical Diseases.

Sincerely,

Kátia Cristina Dantas on behalf of all co-authors

Department of Pathology,

School of Medicine of São Paulo. Av. Dr. Arnaldo 455, 1a andar, sala 1155,

Cerqueira César, São Paulo, SP CEP 01246-903, Brazil.

Phone number: + 55 11 9 9376-9488/+ 55 11 3061-8533

Fax number: +55 11 3062-8098

Email: katia.dantas@fm.usp.br (Dantas, KC).

Email: [kadantas@usp.br](mailto:kadantas@usp.br)

**Responses to reviewers:**

**Reviewer 1**

We appreciate all your comments and suggestions to make the article more complete and clearer.

**Rev 1 - Minor**

1.1. *There are no line numbers in the manuscript. This makes it difficult to track observations.*

**Answer 1.1. Rev1 minor. According to the request of reviewer 1, a continuous number was inserted in the lines.**

1.2. *In the context of identification/characterization, it is better to refer to Cryptococcus rather than cryptococcosis (throughout all the text). Although it is also usable.*

**Answer 1.2. Rev1 minor. According to the reviewer's suggestion 1 we changed cryptococcosis to *Cryptococcus* spp. when appropriate in the text and marked the changes in red.**

**Rev 1. Specific Comments:**

**Rev.1. Abstract**

***Rev 1.1.1. Background***

*1.1. In the introduction, it seems to me that a sentence is missing explaining the gap that the study intends to fill.*

**Answer. Rev.1.1. Background: According to the reviewer's suggestion, we inserted the relevance of the study in the text.**

“Cryptococcosis is an opportunistic infection in immunocompromised individuals and in those living with HIV/AIDS. “This study evaluated a protocol for the early diagnosis of meningitis due to *C. neoformans*, utilizing serum and established molecular techniques.” **(Line -35-36).**

***Rev1.1.2. Methods***

*Rev1.1.2.1. Methodology >>> Methods*

**Answer. Rev1.1.2.1 Methods: According to the reviewer's suggestion, we changed the methodology for methods (Line 37) and marked in red.**

*Rev1.1.2.2. The rapid India ink >>> The direct India ink staining (methods)*

**Answer. Rev1.1.2.2. Methods: According to the reviewer's suggestion, we changed the rapid India ink for the direct India ink staining (Line 39) and marked in red.**

***Rev1.1. 3. Principal Findings (results)***

*Rev1.1.3. Please provide comparative values for sensitivity and specificity of tests*

**Answer. Rev.1.1.3.1. Principal findings (results): According to the reviewer's suggestion, we insert comparative values for sensitivity and specificity of tests are marked in red.**

“The 5.8S DNA-ITS PCR was more sensitive (89-100%) and specific (100%) than the 18S rDNA PCR and conventional tests (India ink staining and latex agglutination) for identification of *C. neoformans*. While the 18S PCR exhibited a sensitivity (72%) similar to that of the latex agglutination assay in serum samples, it was superior to the latex agglutination assay when testing CSF, with a sensitivity of 84%. However, the latex agglutination was superior to the 18SrDNA PCR in specificity in CSF (92%). The 5.8S DNA-ITS PCR yielded the highest levels of accuracy (96-100%) of any test for detection (serological and mycological) of *C. neoformans* in both serum and CSF.” **(Line: 44-51).**

***Rev1.1.4. Conclusion***

*Rev1.1.4. The comparative aspect of the tests proposed as an objective of the manuscript is not clearly expressed in the conclusion.*

*Here a proposal: “Molecular techniques, in particular nested PCR specific to the 5.8Sr DNA sequence, show a better detection of Cryptococcus infection than conventional detection methods and 18S PCR, both in serum and CSF. They also allow a better early identification in case of negative serology and mycology, potentially allowing an early diagnosis”.*

**Answer. 1.4.1. Conclusion: Thank you for the proposal to make the conclusion clearer by highlighting the results. (Line: 53-56).**

“Utilization of the nested 5.8S PCR was superior to other techniques for the diagnosis of cryptococcosis. The possibility of using serum, a non-invasively collected material, in a targeted 5.8S PCR analysis to identify *Cryptococcus* spp. is recommended, especially in immunosuppressed patients.”

***Rev1.2. Introduction***

Rev1.2.1. New data on the global burden of cryptococcosis in HIV patients were recently published in 2022 by Radha Rajasingham et al. https://doi.org/10.1016/ S1473-3099(22)00516-3. Please update

**Answer. Rev1.2.1. Introduction: Thank you for indicating this paper. According to your suggestion, these references were added and updated with the latest data.**

“In 2022, Rajasingham, et al. estimated that there are 152,000 cases (111,000-185,000) of cryptococcal meningitis, resulting in 112,000 CM-related deaths (79,000-134,000) [11]. In 2014 there was a reduction in the estimated global burden of HIV-associated cryptococcal meningitis, probably due to the expansion of antiretroviral therapy. It has been estimated that cryptococcosis accounts for 19% of opportunistic diseases in HIV-positive patients [11]. ". **(Line: 86-91).**

Rev1.2.2.

- **Answer. Rev1. 2.2. Introduction: To clarify reviewer 1's questioning, this paragraph was rewritten. “**In Brazil, studies on neurological diseases in HIV/AIDS patients showed cryptococcosis to be the second leading cause of death, with a mortality rate of 45-65% (toxoplasmosis was the first and tuberculosis was the third most frequent) [13-15].” **(Line: 96-98).**

Rev1.2.3.

*The diagnosis of cryptococcosis is complex and is usually based on a combination of clinical and radiological suspicion followed by confirmation by laboratory diagnosis*

*In patients with HIV-associated infections, culture of cerebrospinal fluid (CSF) is the gold standard for diagnosis of cryptococcal meningitis in viable sample…*

**Answer. Rev1.2.3. Introduction:** **According to reviewer 1's note, this paragraph was rewritten.**

“The diagnosis of *Cryptococcus* spp. is complex and typically involves a combination of symptoms, including headache, fever, cranial neuropathies, mental alteration, lethargy, memory loss and signs of meningeal irritation, confirmed by laboratory testing [17]. Currently, the laboratory diagnosis of *Cryptococcus* spp. is based on mycological, histopathological and serological tests [17-26]. The Brazilian Unified Health System (SUS) provides different methods for the diagnosis of CM. The conventional method for patients with HIV-associated infections is the direct examination of slides stained with India ink and culture of the sample in Sabouraud dextrose agar (SDA). Both tests are the gold standard for the diagnosis of *Cryptococcus* spp. [21-25]. According to Vidal et al. (2018), detection of CrAg LFA in serum, CSF, and whole blood shows great sensitivity and specificity. In this retrospective review, we explored the value of other methodologies, since during the study period; CrAg LFA was not available for analysis [26]. **(Line: 111-123).**

**Rev1.3.0. Methods**

*Rev1.3.1. Although it is of little importance, I would not begin this section with ethical considerations.*

**Answer. Rev1. 3.1. Methods: According to the reviewer's suggestion, the item was changed after the description of the patients.**

*Rev1.3.2. Serum and CSF from the same patient? Two types of samples per patient? Please specify*

**Answer Rev1.3.2. Methods: In order to clarify the doubt of reviewer 1, we performed the analyzes with two samples from the same patient (serum and CSF).**

*Rev1.3.3. The study was approved by the institutional ethics committees of the Hospital das Clinicas da Faculdade de Medicina da Universidade de Sao Paulo - HCFMUSP (No. 0635/2007) and the Instituto de Infectologia Emílio Ribas -IIER (No. 63/2007).*

**Answer Rev1.3.3. Methods:** **The text was edited according to the reviewer's suggestion, (Lane: 147-150). “**Serum and CSF were collected from 49 patients, a total of 98 samples**,** between January 2008 and December 2009 following their admission to the emergency units at Instituto de Infectologia Emílio Ribas (IIER) and Hospital das Clinicas da Faculdade de Medicina da Universidade de Sao Paulo (HCFMUSP) with suspected meningitis”.

*Rev1.3.4. Patients >>> patients and samples*

**Answer Rev1.3.4. Methods**: **The text was edited according to the reviewer's suggestion, (Line 146).**

*Rev1.3.5. After initial isolation in culture subsequent identification was by physiological and biochemical tests as described below: is it possible to quote at least the tests used?*

**Answer Rev1.3.5. Methods:** **After initial isolation in culture subsequent identification was by physiological and biochemical tests (Niger seed agar plates; Christensen's urea medium and subtyped using L-canavanine glycine bromothymol blue (CGB) medium), (Line: 153-156).”**

*Rev1.3.6. and AIDS/HIV-positive and positive for cryptococcosis (group IV; n=18) >>> and AIDS/HIV-positive and positive for cryptococcosis (group IV; n=18)*

**Answer Rev1.3.6. Methods: The text was edited according to the reviewer's suggestion, “***and AIDS/HIV-positive and positive for cryptococcosis (group IV; n=18)”,* **(Line: 162-163).**

*Rev1.3.7. (600-base pair [bp] product) and 18S 9429-bp product) >>> [600-base pair (bp) product] and 18S (9429-bp product).*

**Answer Rev1.3.7. Methods**: **The text was edited according to the reviewer's suggestion, “**[600-base pair (bp) product) and 18S (429-bp product)”**, (Line 231).**

*Rev1.3.8. Multiplex PCR: Cryptococcus spp >>> Cryptococcus spp*

**Answer Rev1.3.8. Methods**: **The text was edited according to the reviewer's suggestion,** “*Cryptococcus* spp.*”,* **(Line 241).**

*Rev1.3.9. On the basis of the drawbacks of conventional routine techniques as notified in the introductory part, would you have the possibility to integrate the detection of inter-ribosomal DNA in the daily practice?*

**Answer Rev1.3.9. Methods**: This approach can be utilized in daily practice on samples from patients who have a clinical suspicion of cryptococcal meningitis or therapy follow-up due to the accuracy of the results obtained and the brief period (two days) used in the PCR test.

*Rev1.3.10. The significance level was set at p ≤ 0.05: please make the font uniform.*

**Answer Rev1.3.10. Methods: The font was standardized according to reviewer 1's suggestion, “**The significance level was set at p ≤ 0.05.” **(Line: 288-289).**

***Rev1.4. Results***

*Rev1.4.1. How were non-cryptococcal agents identified in the samples? other fungi, bacteria and viruses?*

**Answer Rev1.4.1. Results:** The laboratories from the Institute of Tropical Medicine, IIER, and HCFMUSP, carried out the identification of fungi, bacteria, and viruses, according to the Ministry of Health's guidelines. (Line: 306-309).

**(https://bvsms.saude.gov.br/bvs/publicacoes/guia_vigilancia_saude_volume_1.pdf)**

*Rev1.4.2. Table 2: Order of presentation of parameters = underlying diseases first, then HIV status.*

**Answer Rev1.4.2. Results: According to Reviewer 1's suggestion, the order of presentation of the parameters was changed, inserting the underlying diseases first, then the HIV status, in the Table. Table 2 was changed to Table 3. (Line 305).**

*Rev1.4.3. Prior to the Cryptococcus spp. assay, patient samples were subjected to nested PCR against the housekeeping gene GADPH, confirming in all samples the presence of human DNA and the absence of PCR inhibitors: This seems redundant to me because it has already been mentioned in the method section (page 10 - 11). Also, the interpretive explanations of the Kappa test should be in the methods, they are not results.*

**Answer Rev1. 4.3. Results: According to the reviewer's suggestion, the paragraph was deleted, and the interpretive explanations *of the Kappa test* were transferred to the methods. (Line: 292, Table 2).**

*Rev1.4.4. All assays were performed in triplicate and showed 100% reproducibility. In addition,*

**Answer Rev1.4.4. Results: Thanks to the reviewer for this note, text has been moved to session methods” (Line: 238-239).**

*Rev1.4.5. It is essential to use the new nomenclature of Cryptococcus species: Cryptococcus var grubii >>> C. neoformans. This is for the whole manuscript*

**Answer Rev1.4.5. Results: According to Reviewer 1's guidance, the nomenclature was changed to. *C. neoformans* throughout the manuscript and marked in red.**

*Rev1.4.6. The 18S PCR assay results in HIV-positive patients were similar for both sera and CSF, (Kappa index of 0.566 and 0552, respectively). However, when testing CSF, the 18S PCR exhibited a greater accuracy than did the latex agglutination test.*

**Answer Rev1.4.6. Results:** The 18S PCR assay results in HIV-positive patients were similar for both serum (K=0.566; 0.423-0.709; p=0.001) and CSF (K=0.552; 0.403-0.701; p=0.002), and agreement was only moderate. The Kappa index for the HIV-negative patients was 0.746; (0.583-0.909; p=0.001) for serum and 0.876 (0.757-0.995; p<0.001) for CSF, and agreement was substantial. **(Line: 338-342).**

*Rev1.4.7. A comparative analysis of the 18SDNA primers with the latex agglutination assay for sera yielded a similar specificity and sensitivity, suggesting that the latex agglutination assay can be used as a complementary alternative test to this PCR test*

**Answer Rev1.4.7. Results**: **According to the reviewer's suggestion this paragraph was rewritten to improve clarity.** Comparative analysis of serum samples did not demonstrate a difference between the 18SrDNA PCR and latex agglutination assays. These results demonstrate that latex agglutination can detect early-stage *Cryptococcus* spp. In CSF samples from HIV-positive patients, the 18S PCR demonstrated a better performance in terms of sensitivity. The latex agglutination test was the most specific, but the 18S PCR had a high sensitivity, and can be used as a complementary test. **(Line: 342-347).**

*Rev1.4.8. The comparative analysis between conventional (mycological India ink staining and serological). Overall, I note that the proportions compared by test are not presented. This could improve the understanding of some paragraphs.*

**Answer Rev1.4.8. Results: We insert the comparison of proportions to improve understanding, as suggested by the reviewer.** “The comparative analysis between conventional (mycological and serological) and molecular (18S and 5.8S PCR) methods showed that the molecular methods had a higher sensitivity and specificity (Table 4)”, (**Line: 368-370).**

**Rev1. 5. Discussion**

*Rev1.5.1. The increased availability of anti-retroviral treatment (ART) plus aging of the HIV population may have contributed to changes in the spectrum of disease manifestation, including complications that may be attributable to the neurotoxic consequences of ART >>> I'm having trouble finding the connection of this sentence to the previous sentences.*

**Answer Rev1.5.1. Discussion: This paragraph was deleted from the discussion session because this paragraph was outside the scope of this study.**

*Rev1.5.2. In addition, false-positive results in sera and CSF from HIV-positive and HIV- negative patients can occur under various conditions, such as in the presence of rheumatoid factors or infections with Trichosporon beigelii, Klebsiella pneumonia, Capnocytophaga canimorsus or Stomatococcus mucilaginosus. [20, 59].*

**Answer Rev1.5.2. Discussion: Discussion: The paragraph has been rewritten to make it clearer. “**In addition, false-positive results in sera and CSF from HIV- negative patients can be due to cross-reactions with other opportunistic pathogens, such as *Trichosporon beigelii,* *Klebsiella pneumonia*, *Capnocytophaga canimorsus* or *Stomatococcus mucilaginosus*, making diagnostic confirmation difficult [19, 57]. **(Line: 412-416).**

*Rev1.5.3. A comparative analysis of the 18SDNA primers with the latex agglutination assay for sera yielded a similar specificity and sensitivity, suggesting that the latex agglutination assay can be used as a complementary alternative test to this PCR test*

**Answer Rev1.5.3. Discussion: The paragraph has been rewritten to make it clearer.,. “**The comparative analysis demonstrated that the 18SrDNA PCR and latex agglutination assays for early detection of *Cryptococcus* spp. in CSF samples from HIV-positive patients can be complementary. The 18S PCR exhibited better performance in terms of sensitivity [58], while the latex agglutination test was more specific.” **(Line: 429-432).**

*Rev1.5.4. The lower sensitivity and specificity with the 18S primers in the CSF samples may be due to interference in gene amplification by components of the Cryptococcus spp. cell wall or the antiretroviral treatment >>> How do you explain the particularity of these two parameters at the level of 18S primers*

**Answer Rev1.5.4. Discussion:** The study with the 18S and 5.8S primers showed that the 5.8S primer has higher accuracy when compared to the 18S primer. The ITS region (18S, 5.8S and 28S) of rDNA has been the most frequently used region for the detection of fungal sequences due to its high degree of variation compared to other ribosomal DNA regions, which facilitates identification. The detection of *C. neoformans* DNA in CSF samples by PCR has been studied by some groups (Rappelli et al., 1998; Bialek et al., 2002; Evertsson et al., 2000; Mitchell et al., 1994; Prariyachatigul et al., 1996; Paschoal et al., 2004; Saha et al. 2009; Martins et al., 2015). However, few papers report the experience of detection in serum samples. In 2009, Saha et al, showed the efficacy of the 18SrDNA region primer in serum and CSF samples. However, our study showed that the 5.8SrDNA region primer showed higher accuracy both in CSF samples, as already described in the literature by Martins et al., (2015), and in serum samples. One hypothesis is that the CN6 oligonucleotide is a unique region found only in the ITS sequences of the two species of *Filobasidiella* and not in closely related species of the genus *Filobasidium*. And primer CN5 contains more conserved sequences that overlap with the 3' end of the 5.8S rDNA gene. The primer pairs CN5-CN6, are more specific for *C. neoformans*, justifying the higher accuracy.

*Rev1.5.5. Multiplex PCR and sequencing data have confirmed that 95-100% of C. neoformans biological samples from AIDS patients worldwide, including in Brazil, are from the grubii strain (serotype A) >>> please consider the new nomenclature*

**Answer Rev1.5.5. Discussion: Nomenclature has been revised throughout the manuscript and marked in red.**

*Rev1.5.6. The present study has limitations that must be acknowledged. The patients’ medical records did not contain all relevant information, such as occupational risk factors. In addition, the analysis of patients’ follow-up was limited to their hospitalization and so the risk of recurrence or other complications after discharge could not be assessed and the small??? >>> truncated sentence?*

**Answer Rev1.5.6. Discussion**: **The paragraph has been rewritten to make it clearer.** “A limitation of the present study was the sample size since the number of HIV-negative patients was lower compared to the HIV-positive subjects. This reduced the power of the statistical evaluation. In addition, patients’ medical records did not contain all relevant information, such as occupational risk factors. In addition, the analysis of patients’ follow-up was limited to their hospitalization and so the risk of recurrence or other complications after discharge could not be assessed. Lastly, as the study was retrospective, it was not possible to consider the test lateral flow assay cryptococcal antigen (CrAg LFA) when testing these samples**.**” **(Line: 456-463).**

*Rev1.5.7. Although further studies are needed, our results indicate that the combination of nested 5.8S PCR testing in association with the latex agglutination test may increase the ability to diagnose and monitor cryptococcosis in patients.*

**Answer Rev1.5.7. Discussion: According to our results, nested 5.8SrDNA does not need any other complementary methodology. So, this paragraph has been rewritten.**

Our results indicate that the nested 5.8S PCR can increase the ability to diagnose cryptococcosis, and we suggest its use to monitor patients in the future. Furthermore, the possibility of using serum, a non-invasively collected material, in a targeted 5.8S PCR analysis to identify *Cryptococcus* sp. is worth highlighting, especially in immunosuppressed patients. **(Line: 464-468).**

**Reviewer 2**

We thank you for all the suggestion, which were promptly addressed and added much to this manuscript. All suggested articles were properly inserted in our introduction.

*Rev2.1. Methods. The study aims evaluated the performance of molecular methods in the diagnosis of cryptococcal meningitis and was clearly stated. The design and population are adequate. This review is not sure the sample size is sufficient and recommend a statistician to review this issue. no concerns about ethical or regulatory requirements.*

**Answer Rev2. 1: Methods.** The sample size is based on a review histology of cases of death by *Cryptococcus* that occurred between 1954 to 2015 in the Hospital das Clínicas da FMUSP. Considering ten years (1998 to 2007), before the beginning of the insertion of patients in the present work found 50 cases of cryptococcosis, which means five death/per year, with a mortality rate varying between 45 - 65%, assuming mortality rate of 45% had 11.33% cases/year, similar to N including, 25 cases in two years. According to the Brazilian consensus report on cryptococcosis in 2008; Journal of the Brazilian Society of Tropical Medicine 41(5):524-544, Sep-Oct, 2008), and Diamond RD, Bennett JB. Prognostic factors in cryptococcal meningitis. A study of 111 cases. Annals of Internal Medicine 80:176-181, 1974.

**Rev2.2.** Conclusions. The conclusions are supported by the data presented, but the main concern is related to serum used to obtain colonies of *Cryptococcus*, which is a unreliable method for this aim. *This review did not find the description of limitations of analysis, supposed to be written by the authors. There are some discussions about the utility of the obtained data to address the cryptococcal meningitis diagnosis.* The public health relevance was demonstrated.

**Answer Rev2.2: Conclusions.:** To clarify reviewer 2's doubts about the conclusions, we have rewritten them.

In abstract:

Utilization of the nested 5.8S PCR was superior to other techniques for the diagnosis of cryptococcosis. The possibility of using serum, a non-invasively collected material, in a targeted 5.8S PCR analysis to identify *Cryptococcus* spp. is recommended, especially in immunosuppressed patients.

**(Line.: 53-56).**

Final conclusion:

A limitation of the present study was the sample size since the number of HIV-negative patients was lower compared to the HIV-positive subjects. This reduced the power of the statistical evaluation. In addition, patients’ medical records did not contain all relevant information, such as occupational risk factors. In addition, the analysis of patients’ follow-up was limited to their hospitalization and so the risk of recurrence or other complications after discharge could not be assessed. Lastly, as the study was retrospective, it was not possible to consider the test lateral flow assay cryptococcal antigen (CrAg LFA) when testing these samples**. (Line.: 456-463).**

“The serum was only tested by serological and molecular tests, since the purpose of the study is to use a test using non-invasive collection, mainly in immunosuppressed patients. Given this, molecular techniques, in particular nested PCR specific for the 5.8Sr DNA sequence, demonstrated better detection of *Cryptococcus* infection than conventional detection methods and 18S PCR, both in serum and CSF, as well as in the serological test, potentially allowing an early diagnosis.”

**Rev2.3. Summary and General Comments**

**Rev2. 3.1.:** 1. The objective of this study was to perform a comparative analysis of the sensitivity, specificity, and accuracy of conventional and molecular diagnostic methods for the detection of *Cryptococcus neoformans* in serum and cerebrospinal fluid (CSF) from individuals with meningitis. *Although interesting, there are several concerns that need to be addressed with this manuscript. The major concern is related to culture of serum to obtained Cryptococcus colonies (CSF and clotted blood samples were centrifuged….and the pellet fraction was cultured on SAB agar)*.

**Answer Rev2.3.1.:** We thank reviewer 2 for his concern with how we describe the processing of biological samples. Mycological assays were rewritten in Methods (Mycology session: Lane.: 208-215). The culture was performed only from CSF samples, and the serum sample was processed and analyzed by serological and molecular tests. And in this study the aimed to propose the early diagnosis of meningitis due to *C. neoformans*, providing the patient with the minimally invasive collection, that is, serum and use molecular techniques already established in the literature.

*Rev.2.3.2. 2. This is neither a conventional nor a recommended method. Blood cultures are preferred to serum for cultivation of fungi. It is not clear the specimen used to obtain positive cultures is many sentences (e.g. Cryptococcus spp. were confirmed in 25 cases by isolation in culture or The 5.8S PCR exhibited the highest sensitivity and specificity with an accuracy of 100%; the Kappa index analysis showed almost perfect agreement with the gold standard (culture) in serum samples (0.918). This issue must be strictly defined because there are many possible interpretations of the results.*

**Answer Rev2.3.2**.: In order to clarify and avoid misinterpretations, the samples that were analyzed in culture media were only CSF samples. The samples that did not present isolation of *Cryptococcus* sp. were confirmed by histopathological analysis.

“**Processing of samples**. A standard method was followed for the processing of samples [37]. CSF was centrifuged at 1000 g for 15 min (Eppendorf 5810R, Hamburg - Germany). The pellet was used for investigation by culture, direct microscopy (India ink wet mount, Gram stain) and PCR, while the supernatant was tested by serology (latex agglutination analysis) and molecular (18S and 5.8SrDNA) analysis. All samples were tested undilution. Clotted blood was centrifuged at 1000 rpm. for 5 min to obtain serum [37] and subjected to serological (latex agglutination) and molecular (18S and 5.8SrDNA) analyses.

CSF was cultivated on SAB agar [Difco Laboratories, Detroit, MI] at 25ºC and the isolates obtained were analyzed by physiological and biochemical tests consisting of the production of phenol oxidase in subculture on Niger seed agar plates (*Guizotia abyssinica*), as described by Kwon-Chung and Benne and incubated at 37 ⁰C for two weeks [8] and, for urease detection using Christensen's urea medium (Becton Dickinson, New Jersey, USA) [8] and subtyped using L-canavanine glycine bromothymol blue (CGB) medium (Difco Laboratories, Detroit, MI) [37, 38]. After identification, the isolates were stored in 80% glycerol (Sigma-Aldrich, St. Louis, MO, USA) at -80°C”. **(Line 195-209).**

Rev.2.3.3. The authors should explain why they choose latex agglutination instead of lateral flow assay, the method used more extensively for immunological diagnosis of meningeal Cryptococcosis.

**Answer Rev2.3.3.:** According to Vidal et al. (2018), CrAg LFA in serum, CSF and whole blood showed great sensitivity and specificity. By streamlining diagnostic tests, the use of this instrument can reduce expenses. In this retrospective review, we explore other methodologies; because during the study period, CrAg LFA was not available for sample analysis.

*Rev.2.3.4. The English style deserves an extensive revision.*

**Answer Rev2.3.4: The manuscript was submitted to professional language editing (see attached certificate).**

*Rev.2.3.5. Additional comments: Abstract: The 5.8S DNA-ITS PCR yielded the highest levels of accuracy of any test? Or among the tested methods? The correct sentence may be “negative serology and mycology results”.*

**Answer Rev2.3.5.:** “The 5.8SrDNA-ITS was more accurate than the other methods tested (serological, mycological, and 18S PCR assay). The results showed 100% in CSF samples, and 94% in serum samples, the lower accuracy in serum is due to the group of HIV-positive patients because two patients of Cryptococcus sp. only were detected in CSF”.

**Rev. 2.4. Introduction section:**

Rev.2.4.1. Please, revise the data because fungi are not a major contributor to opportunistic infections in those with advanced HIV infection. The number of cases and death attributable to cryptococcal meningitis should be updated.

A reference could be Rajasingham R, Govender NP, Jordan A, et al. The global burden of HIV-associated cryptococcal infection in adults in 2020: a modelling analysis [published online ahead of print, 2022 Aug 29] [published correction appears in Lancet Infect Dis. 2022 Oct 31]. Lancet Infect Dis. 2022; S1473-3099(22)00499-6. doi:10.1016/S1473-3099(22)00499-6

**Answer Rev2.4.1. Introduction: Thank you for indicating this paper. According to your suggestion, these references were added and updated with the latest data .**

“In 2022, Rajasingham, et al. estimated that there are 152,000 cases (111,000-185,000) of cryptococcal meningitis, resulting in 112,000 CM-related deaths (79,000-134,000) [11]. In 2014 there was a reduction in the estimated global burden of HIV-associated cryptococcal meningitis, probably due to the expansion of antiretroviral therapy. It has been estimated that cryptococcosis accounts for 19% of opportunistic diseases in HIV-positive patients [11]."**(Line: 86-91).**

*Rev. 2.4.2. The following information needs a reference “…indirect cases obtained from the STD/HIV-AIDS Department of the Brazilian Ministry of Health.” “Use initial capital letter for Cryptococcosis and other mycoses throughout the text”. “The following reference on PCR-based diagnosis could be added: Martins Mdos A, Brighente KB, Matos TA, Vidal JE, Hipólito DD, Pereira-Chioccola VL. Molecular diagnosis of cryptococcal meningitis in cerebrospinal fluid: comparison of primer sets for Cryptococcus neoformans and Cryptococcus gattii species complex. Braz J Infect Dis. 2015;19(1):62-67. doi:10.1016/j.bjid.2014.09.004 Clarify the idea of “reducing patients’ risk of progression” for improve understanding”.*

**Answer Rev2.4.2. Introduction. Thank you, reviewer, 2 for all the suggestions on how to improve the introduction and make the text clearer. References have been inserted in the text. And it was revised throughout the text whenever Cryptococcosis was cited it was capitalized. Regarding “reducing patients’ risk of progression” in the text was rewritten in order to make the text clearer.**

“The examination of cases from regional centers or from the STD/HIV-AIDS Department of the Ministry of Health form the foundations for the diagnosis of the infections [17]”. **(Line: 103-105).**

Departamento de Doenças de Condições Crônicas e Infecções Sexualmente Transmissíveis – MINISTERIO DA SAUDE. Boletim Epidemiológico – HIV Aids. Julho de 2017 a junho de 2018; 2018, <http://www.aids.gov.br/pt-br/pub/2018/> boletim-epidemiologico-hivaids-2018; [accessed 13 March 2019].”

**“**In 2015, Martins et al suggested that the CN4/CN5 primer set amplified gene sequences encoding rDNA and was highly sensitive for the identification of *C. neoformans*/*C. gattii* in CSF from patients with clinical suspicion of cryptococcal meningitis [34]. **(Line: 132-136).**

**Rev2.5. Methods section**

*Rev2.5.1. Abbreviations and acronyms are often defined the first time they are used within the abstract and again in the main text and then used throughout the remainder of the manuscript. Please consider adhering to this convention (e.g. IIER and HCFMUSP).*

**Answer Rev2.5.1. Methods:** **According to the review guidelines, abbreviations and acronyms were revised throughout the text considering IIER and HCFMUSP and it was mark in red.**

*Rev2.5.2. There are also mistakes such as ml instead of mL and l instead of L (liter). Please, revise the entire manuscript.*

**Answer Rev2.5.2. Methods:** **The errors pointed out have been corrected throughout the text and it was mark in red.**

*Rev2.5.3. It is not clear how many samples were collected from each patient. Prefer Candida spp., instead of Candida sp.*

**Answer Rev2.5.3. Serum and CSF were collected from 49 patients, with a total of 98 samples. It has been corrected throughout the text by changing it to *Candida* spp and it was marked in red.**

*Rev2.5.4. Control strains section: The entire paragraph should be reworded to improve understanding. Use square brackets for: H. capsulatum, ATCC 28308 (CDC: B973), ATCC 12700 (CDC: A811), and HC200 (GenBank: DQ239887) and aspergillosis (Aspergillus spp)*

**Answer Rev2.5.4. Methods: This paragraph was** **rewritten Specificity of molecular methods were evaluated using isolates well characterized by the ATCC and CDC.** “They consisted of paracoccidiodomycosis (*P. brasiliensis* - 18 and B-339 ATCC 32069), nocardiosis (*Nocardia* spp.), candidiasis (*Candida albicans* and *C. parapsilosis*), histoplasmosis [*H. capsulatum,* ATCC 28308 (CDC: B973), ATCC 12700 (CDC: A811), and HC200 (GenBank: DQ239887)] and aspergillosis (*Aspergillus* spp.).” **(Line: 183-186).**

*Rev2.5.5.*

*2.5.5.a. Mycology section: Is it correct: clotted blood samples were centrifuged? What the purpose?*

*2.5.5.b. Nowadays, L-canavanine glycine bromothymol blue (CGB) medium is unsuitable for subtyped the species complexes of Cryptococcus.* Molecular identification of the isolate is highly desirable.

**Answer Rev2.5.5. Methods:**

**2.5.5.a. This paragraph has been rewritten “Processing of samples**. A standard method was followed for the processing of samples [37]. CSF was centrifuged at 1000 g for 15 min (Eppendorf 5810R, Hamburg - Germany). The pellet was used for investigation by culture, direct microscopy (India ink wet mount, Gram stain) and PCR, while the supernatant was tested by serology (latex agglutination analysis) and molecular (18S and 5.8SrDNA) analysis. All samples were tested undiluted. Clotted blood was centrifuged at 1000 rpm. for 5 min to obtain serum [37] and subjected to serological (latex agglutination) and molecular (18S and 5.8SrDNA) analyses.**” (Line 195-201)**

2.5.5.b. L-canavanine glycine bromothymol blue (CGB) medium was used in routine diagnostics during the collection period; however, we emphasize molecular tests to confirm the species.

*Rev2.5.6.*

*2.5.6.a. Nested PCR section Please, revise the marks: (600-base pair [bp] product) and 18S 9429-bp product). Also in Table 1*

*2.5.6.b. Multiple PCR section Use non-italics for spp. Revise all text*

**Answer Rev2.5.6. Methods:**

**2.5.6.a. It has been corrected in the Nested PCR section (600 base pair [bp] product) and 18S product (429-bp), Line 231 and in Table 1.**

**2.5.6.b. The multiplex PCR section has been corrected throughout the text by not using multiplex PCR in italics.**

*Rev2.5.7. Sequencing Analysis section: The sentence is repetitive: To avoid contamination of components, preparation of reaction mixtures and addition of template DNA were performed in separate rooms.*

**Answer Rev2.5.7. Methods:** **Repeated phrase deleted in sequencing analysis section**

**"to avoid contamination of the components, the preparation of the reaction mixtures and the addition of template DNA were carried out in separate rooms".**

**Rev2.6. Results Section**

*Rev2.6.1. This entire paragraph can be eliminated as it was already presented in Methods section.*

**Answer Rev2.6.1. Results: According to the reviewer’s suggestion, these sentences were deleted**

*Rev2.6.2. Regarding data presented in Table 2, the significance and impact on the Results have not been addressed. Otherwise, they are useless.*

**Answer Rev2.6.2. Results:** The demographic variables: gender, age and ethnicity, of both groups analyzed, showed a homogeneous profile that demonstrates that they do not interfere in the process of infection by *Cryptococcus* sp

*Rev2.6.3.* *The sensitivity was 1 cell/ml for CSF, 10 cells/ml for serum and 1 cell/ml for culture isolates. These procedures for these points are not well explained in the Methods section.*

**Answer Rev2.6.3. Results:** Analysis of the 18S and 5.8S (rDNA-ITS) sequence-specific nested PCR for the detection of *C. neoformans* and *C. gattii* in serum, CSF and cell suspensions of culture isolates yielded a sensitivity of 1 cell/mL for CSF and 10 cells/ml for serum.

*Rev2.6.4. The first letter of multiplex is low capter.*

**Answer Rev2.6.4. Results: According to the reviewer’s suggestion, *the first letter of multiplex was changed for low capter*.**

*Rev2.6.5. This sentence does not make sense: Serum samples from two HIV-positive patients who were cryptococcal-negative yielded P. brasiliensis and H. capsulatum meningitis. Would be good to clarify what “both groups” refers to in: Two samples from patients in both groups yielded false positive results for cryptococcosis, being instead positive for bacterial meningitis.*

**Answer Rev2.6.5. Results: According to the reviewer’s suggestion this sentence was rewritten.”** The latex agglutination test in the seropositive patients yielded four false-positive tests for *Cryptococcus* spp., subsequently identified by molecular methods as *P. brasiliensis,* *H. capsulatum* and bacterial meningitis.”**, (Line 329-331).**

*Rev2.6.6. Table 3. Inform the number of samples used for each analysis*

**Answer Rev2.6.6. Results:** **According to the reviewer’s suggestion the total number of samples was informed in Table 4.**

*Rev2.6.7.* The following methods were employed for Cryptococcosis diagnosis and not for identification of *Cryptococcus* spp. as informed: To further identify the preferred method for identification of *Cryptococcus* spp., we compared the results obtained by analysis of the 18S and 5.8S PCR-generated DNA sequences with results of latex agglutination and India ink staining.

**Answer Rev2.6.7. Results:** **According to the reviewer’s suggestion, this sentence was rewritten** “In order to select a highly accurate methodology for the diagnosis of Cryptococcosis, we compared the results obtained by analysis of the 18S and 5.8S PCR-generated DNA sequences with results of the latex agglutination test and India ink staining”. **(Line: 355-357).**

**Rev2.7. Discussion Section**

*The following paragraphs are out of scope:*

*In our study of patients with meningitis, a diagnosis of cryptococcosis was confirmed in 51% of the cases, 72% of whom were HIV-positive and progressed to the disseminated form of cryptococcosis. This was consistent with prior findings [3, 11-12, 46-47] and agreed with the study of Yoon et al. [48]. The increased availability of anti-retroviral treatment (ART) plus aging of the HIV population may have contributed to changes in the spectrum of disease manifestation, including complications that may be attributable to the neurotoxic consequences of ART.*

*In both cryptococcosis positive and negative individuals, we observed a higher percentage of males in the cases with meningitis, according to Montoya et al., [49] one of the hypotheses may be hormonal, because testosterone increases the rate of melanin formation in C. neoformans cell and this may enhance pathogenesis [7, 36] This review did not consider limitations of the study were absence of information on the occupational risk factors or the risk of recurrence or other complications after discharge.*

**Answer Rev2.7. Discussion: According to the reviewer’s suggestion, these phrases were deleted as they were not part of the scope of the manuscript.**

**Reviewer 3**

We appreciate all your comments and suggestions to make the article more complete and clearer.

**Rev3 Minor**

*Rev3.1. Methodologies listed were through and fulfilled necessary experimental control conditions. Comparison of the nested and multiplex PCR approaches to gold standard of CSF and Latex agglutination were key to interpretation whether these PCR from sera methodologies would be better or equal in detection of Cryptococcosis. Power analysis was not conducted, or at least not shown to determine if total of 49 patient samples were sufficient with individual groups ranging from n=18 to n=7 values. Groups being tests though are appropriate to address the hypothesis being tested at least with the group AIDS/HIV+ with cryptococcosis having the largest population size (n=18) for comparing across different methodologies. All ethical and regulatory requirements were conducted appropriately as the study was approved by the appropriate committees and patients signed appropriate consent forms.*

**Answer Rev3.1. Methods: According to the reviewer's suggestion, we inserted the confidence interval of the Kappa index in Table 4 and the calculated sample size including patients with cryptococcosis confirmed by isolation in culture and/or histopathological analysis. The researcher responsible for the statistical analysis of this study showed that the analyzed sample is sufficient for the proposed objective. Fifty cases of death from cryptococcosis were confirmed in a tertiary hospital at HCFMUSP, registering an average of 5.1 deaths from cryptococcosis/year similar to our study in the period studied. This study can be proven in the Brazilian consensus report on cryptococcosis in 2008 (Revista da Sociedade Brasileira de Medicina Tropical 41(5):524-544, Sep-Oct, 2008); in the retrospective study Dantas et al., 2021 (Sci Rep (2021), 11:3943. DOI: 10.1038/s41598-021-83587-1 and in other studies Diamond RD, Bennett JB. Prognostic factors in cryptococcal meningitis. A study of 111 cases. Annals of Internal Medicine 80:176-181, 1974).**

*Rev3.2. The results section detailing the findings need to be supplemented with additional data showing replication of values projected as substantial in the accuracy and strength of agreement findings calculated by the inner rater agreement, Landis, and Koch-Kappa Benchmark Scale. Additionally, more clarification needs to be made on how the sensitivity and specificity differences were analyzed rather than a brief citation of previous methodologies.*

**Answer Rev3.2. Results: According to the reviewer's suggestion, additional data regarding the agreement and reliability of the tests were inserted in Table 4. Despite all analyzes showing, significance level p <0.05. In the case of HIV-negative patients, despite the Kappa index showing moderate reliability, the casuistry may not have been sufficient to guarantee this concordance profile. During the study, the casuistry we obtained was higher in HIV-positive patients, due to the incidence of Cryptococcus being higher in this group.**

Rev3.3: Public health relevance is addressed well here in this study but conclusions from the results again must be supported with additional supplemental data from the executed studies showing the values from the patients and their ranges deemed as substantial. Limitations of analysis are moderately clear.

**Answer Rev3.3. Conclusion:** **According to the reviewer's suggestion, the conclusions of the results and the paragraph about the limitations were rewritten supported by some more studies.**

“A limitation of the present study was the sample size since the number of HIV-negative patients was lower compared to the HIV-positive subjects. This reduced the power of the statistical evaluation. In addition, patients’ medical records did not contain all relevant information, such as occupational risk factors. In addition, the analysis of patients’ follow-up was limited to their hospitalization and so the risk of recurrence or other complications after discharge could not be assessed. Lastly, as the study was retrospective, it was not possible to consider the test lateral flow assay cryptococcal antigen (CrAg LFA) when testing these samples**.” (Line 456-463).**

**Rev3.4. Minor Revision**

Rev3.4. Grammatical and sentence structured need to be fixed in the discussion and results sections. Some sentences are incomplete or run on to create confusing attempts at final conclusions. These are minor edits though that can be addressed quickly.

**Answer. Rev3.4. Minor Revision: According to the reviewer's suggestion, this sentence was rewritten, and we corrected grammatical structure and sentences in the discussion and results sections. Some paragraphs, at the suggestion of the reviewers, were excluded because they did not add to the manuscript.**

**The manuscript was submitted to professional language editing (see attached certificate).**
