# Supplementary material for: Comparative analysis of diagnostic methods for the detection of Cryptococcus neoformans meningitis
Source: PLoS Negl Trop Dis. 2023 Mar 6;17(3):e0011140. doi: 10.1371/journal.pntd.0011140 (PMC10019727; doi:10.1371/journal.pntd.0011140)
Supplement: Attachment — Submitted filename: PLOS NTD article Reading Report 2.docx [file pntd.0011140.s003.docx]

**PLOS NTD**

**Article Reading Report entitled: “Comparative analysis of diagnostic methods 1 for the detection of *Cryptococcus neoformans* meningitis”**

Second revision of an interesting article on the identification of the ideal strategy for the early detection of cryptococcosis in serum and CSF samples, minimizing diagnostic time, hospital costs, and most importantly, reducing the progression risk of the infection in the undiagnosed patient.

I want to emphasize here that this second reading is much smoother than the first.

Minor comments are presented in the following lines.

**Abstract**

- Cryptococcosis is an opportunistic infection in immunocompromised individuals and in those living with HIV/AIDS **>>>** Cryptococcosis is a devastating opportunistic infection in immunocompromised individuals, primarily in people living with HIV/AIDS. **L34-5**
- … utilizing serum and established molecular techniques >>> … utilizing established molecular techniques from serum and CSF samples. **L36**
- About the notion of diagnostic costs mentioned in the author's summary section: I didn’t see any other information in the discussion (or results). Can you support the idea that the genetic technique based on the 5.8 TSI sequence can reduce the cost of diagnosis?
- *Cryptococcus neoformans*, the cause of cryptococcosis … >>> *Cryptococcus neoformans*, the main cause of cryptococcosis … **L81**
- It has been estimated that cryptococcosis accounts for 19% of opportunistic diseases in HIV-positive patients >>> It has been estimated that cryptococcosis accounts for 19% of AIDS-related deaths??? **L90-1**
- Both tests are the gold standard for the diagnosis of *Cryptococcus* sp.>>> As far as we know, only culture is the gold standard. Please correct this. **L118-9**
- Dear authors, could you please clarify the number of patients from whom the samples were collected? In the abstract, 39 Brazilian patients with meningitis are mentioned (**L40**) while in the method section 49 patients are reported (**L147**).
- CSF was cultivated on SAB agar [Difco Laboratories, Detroit, MI] at 25ºC and the isolates obtained were analyzed by physiological and biochemical tests consisting of the production of phenol oxidase in subculture on Niger seed agar plates (Guizotia abyssinica) >>> Various biochemical tests on *Cryptococcus* do not produce phenol oxidase at all, but simply exploit its biochemical activity. Please correct this. **L202-5**
- Serum samples were subjected to latex agglutination analysis >>> Serum and **CSF** samples were subjected to latex agglutination analysis? **L212**
- **L250-61**: Redundant information, already presented in **lines 228-47**.
- These tests (multiplex PCR and sequencing) confirmed that 77.4% of the isolates were *C. neoformans* >>> what about the other % (22.6%)? **L320**
- The findings suggest the absence or reduced burden of *Cryptococcus* spp. outside the lung. **L411-2**. Could you explain please the meaning of this sentence in this paragraph?
- One hypothesis for the lower sensitivity and specificity of 18S primers in CSF samples may be interference in the gene amplification process by cell wall components of *Cryptococcus* spp. or even due to interference in the treatment being applied >>> It difficult to understand the basis of these arguments. After extraction and purification of *Cryptococcus* DNA, what component of the cryptococcal wall could interfere with gene amplification? I would just like to learn. Also about interfere with the treatment? ... **P432-35**
- Patients with AIDS >>> patients with HIV/AIDS. **L386.**

The context of the sentence refers more to HIV infection globally, not necessarily to the state of AIDS.

- The nested PCR specific for the 5.8Sr DNA sequence was shown to be superior to both conventional detection methods and 18S PCR for *Cryptococcus* detection. **L472-3**

Could you please specify the parameters by which this superiority was assessed? There are provided in the results and discussion but I feel it is also important to mention them without any details in the conclusion of the manuscript.
